# Natural Selection Mediated Association of the Duffy (*FY*) Gene Polymorphisms with *Plasmodium vivax* Malaria in India

**DOI:** 10.1371/journal.pone.0045219

**Published:** 2012-09-21

**Authors:** Anita Chittoria, Sujata Mohanty, Yogesh Kumar Jaiswal, Aparup Das

**Affiliations:** 1 Division of Genomics and Bioinformatics, National Institute of Malaria Research, New Delhi, India; 2 Department of Biotechnology, Jaypee Institute of Information Technology, Noida, Uttar Pardesh, India; 3 School of Studies in Biotechnology, Jiwaji University, Gwalior, Madhya Pradesh, India; Instituto de Higiene e Medicina Tropical, Portugal

## Abstract

The Duffy (Fy) antigens act as receptors for chemokines as well as for *Plasmodium vivax* to invade human RBCs. A recent study has correlated the occurrence of the FY*A allele of Duffy gene with decreased susceptibility to vivax malaria, but no epidemiological correlation between the distribution of FY*A allele and incidences of vivax malaria has been established so far. Furthermore, if such correlations exist, whether natural selection has mediated the association, is an important question. Since India is highly endemic to *P. vivax* malaria with variable eco-climatic and varying vivax malaria epidemiology across different regions, such a question could well be answered in Indians. For this, we have genotyped the *FY* gene at the −33^rd^ and the 125^th^ nucleotide positions in 250 Indians sampled from six different zonal plus one tribal population covering the whole of India and studied possible correlations with eco-climatic and vivax malaria incidences. No FY*O allele was found, however, both the FY*A and FY*B alleles forming FY*A/FY*A, FY*A/FY*B and FY*B/FY*B genotypes were widely distributed among Indians. Five out of seven population samples significantly deviated from the Hardy-Weinberg equilibrium expectation, and two alleles (FY*A and FY*B) and the homozygote genotype, FY*B/FY*B were clinally distributed over the population coordinates. Furthermore, vivax malaria incidences over the past five years were significantly negatively and positively associated with the frequencies of the FY*A and FY*B alleles, respectively. The Northern Indians were highly differentiated from the other zonal population samples at the *FY* gene, as evidenced from the reconstructed Neighbor-Joining phylogenetic tree. The results specify the role of natural selection in the distribution of *FY* gene polymorphism in India. Furthermore, the hypotheses on the part of the FY*A allele in conferring protection to vivax malaria could be validated following population genetic studies in a vivax malaria epidemiological setting, such as India.

## Introduction

The human Duffy blood-group (Fy) antigens are major determinants for the invasion of malaria parasite *Plasmodium vivax* in RBCs and are encoded by Duffy (*FY*) gene which is characterized by three different alleles at the genetic level; FY*A, FY*B and FY*O [1]–[3]. While the FY*A and FY*B alleles could be identified by a missense nucleotide mutation (G125A) and present the common Fy(a+b−), Fy(a−b+) and Fy(a+b+) serological phenotypes, the FY*O allele (absence of the Fy antigen) gives rise to Fy(a−b−) phenotype due to T-33C mutation at the promoter region of the *FY* gene [4]. Since the T-33C mutation stops the expression of the *FY* gene and thus blocks the red blood cell invasion of the human malaria parasite *Plasmodium vivax*; the FY*O allele is at or near to fixation in most of the sub-Saharan African populations, with very rare occurrence in populations outside Africa - indicating the role of positive natural selection for the FY*O allele in humans [5], [6]. This argument is in agreement with the observation of resistance to *P. vivax* infection in humans homozygous to the FY*O allele [7] with some exceptions [8]. Very recently, the FY*A allele has been shown to significantly diminish binding of *P. vivax* Duffy binding protein in comparison to FY*B, hence it is argued that individuals possessing the FY*A allele are in reduced risk to *P. vivax* malaria [9]. However, no correlation between the allele frequency and vivax malaria epidemiology has yet been established in any endemic country setting. Now-a-days, Duffy blood group is majorly determined by DNA sequencing of the *FY* gene [10], which enables in identification of any of the three different genotypes (FY*A/FY*A, FY*A/FY*B and FY*B/FY*B) in a single individual. Population data from multiple individuals further enables in determination of the frequency of three different alleles (FY*A, FY*B and FY*O) which could be utilized in evolutionary inference of the *FY* gene [5], [6]. Since eco-climatic factors are highly variable in India (large longitudinal and latitudinal transects) with complex malaria epidemiology due to both *P. vivax* and *P. falciparum* infections [11] and Indians are genetically heterogeneous with complex population history [12], [13], [14]; we were interested to determine (i) the variable distribution of *FY* gene polymorphisms and genetic differentiations among populations living in different eco-climatic conditions, and (ii) possible correlation between *FY* gene polymorphisms and vivax malaria epidemiology by population genetic analyses in India. Although previous studies have indicated the distribution of different serological phenotypes of the Fy antigen in different local populations in India [15], [16], [17], [18], neither any survey on the distribution of different *FY* alleles representing the whole of India has been conducted, nor any correlation of *FY* polymorphisms to eco-climatic and/or vivax malaria epidemiology has been established. In addition, the results on the role of the FY*A allele to reduced risk of malaria in comparison to the FY*B allele [9], has not been tested/reconfirmed in any field vivax malaria epidemiological settings so far. We propose that no better place other than India could serve as the model field to understand evolution of such host-parasite interaction [19], with vivax malaria as an example.

## Materials and Methods

In order to catch most of the polymorphisms of the *FY* gene and to meaningfully conduct population genetic survey, we have divided the whole of India into six distinct zones; *viz.*, North India (NI), Central India (CI), West India (WI), South India (SI), East India (EI) and North-East India (NEI). Further, since many tribes live in different Indian states, we have included samples from three Indian tribes; Juango, Bonda and Kutia Kandha inhabiting in Odisha state. The details of the Indian states included under each zone and the number of individuals sampled from each state are provided in the Table S1. In total, 250 healthy and unrelated Indians (including 28 tribal individuals) representing almost all the political states of India were sampled. For positive control, blood samples from two Africans (Cameroonians colleagues of the authors working in India) were also analyzed. From each individual, two milliliter of intravenous blood was collected in heparinized vacutainers and stored at −20°C until DNA extraction was performed.

We have selected the 1096 bp region of the *FY* gene located in the human chromosome 1 (Figure S1), comprising the two main polymorphic sites which govern the FY*O allele (T-33C) in the promoter region and the FY*A/FY*B alleles (G125A) in the exon-2. For ease in PCR amplification and automated DNA sequencing, we have divided the 1096 bp region into two overlapping sequences (Figure S1) employing two primer pairs (Table S2). Genomic DNA from blood sample of each individual was extracted and PCR amplification reactions were carried out following standard protocols in a final volume of 20 µl. Five microliters of amplified PCR product of each fragment were run on 2% agarose gel to check the quality of amplification. If a single band without any primer-dimer was present, the amplicons were considered for DNA sequencing. For this, the PCR amplicons were purified with Exonuclease-I and Shrimp Alkaline Phosphatase (Exo-Sap, Fermentas, Life Sciences) following standard protocol. Sequencing reactions were followed with Big Dye Terminator (BDT) ready reaction mix as per the Applied Biosciences (ABI) protocol and DNA sequencing was performed in an ABI 3730XL DNA Analyzer (in-house facility of NIMR). Each DNA fragment was sequenced in both the forward and reverse directions (2× coverage) and the resulted DNA sequences were assembled and edited using the SeqMan and EditSeq computer programs (DNASTAR, Madison, WI, USA) for each individual. The DNA sequence chromatograms were carefully visually inspected for the occurrence of single/double peaks in the sequence chromatogram at both the T-33C and G125A nucleotide positions. Altogether, 252 DNA sequences (250 Indian, two African) were aligned using the MegAlign computer program (DNASTAR) following Clustal-W algorithm to identify Single Nucleotide Polymorphisms (SNPs) at these two nucleotide positions (T-33C and G125A). For instance, detection of the C nucleotide in a single peak at the −33^rd^ position indicates the presence of FY*O homozygote, both C and T in double peak as heterozygote genotype and the T nucleotide in a single peak indicates the absence of FY*O genotype. Similarly, detection of the G and A nucleotides (in single peaks) at the 125^th^ position indicate homozygosity for FY*A and FY*B, respectively, whereas presence of double peaks of both G and A nucleotides at this position categorize the individual as a heterozygote (FY*A/FY*B). Following these approaches, each of the 250 Indians and the two Africans were independently genotyped and frequencies of each allele for the seven Indian population samples were calculated based on the genotype data for population genetic analyses (see below).

The genotype data (FY*A/FY*A, FY*A/FY*B and FY*B/FY*B) from each of the seven zonal population samples were analyzed by Chi-square Test to determine if the population samples deviate from the expectation under the Hardy-Weinberg (H-W) equilibrium. Genetic differentiation between pairs of population samples were determined by two test statistics; (i) Fisher's Exact Test of differentiation using the Arelquine computer program (http://cmpg.unibe.ch/software/arlrquin3), and (ii) Nei's genetic distance between a pair of population sample (as measured by *D*) [20] using the POPGENE computer program (http://www.ualberta.ca/~fyeh/). In order to visualize genetic interrelationships among different Indian population samples, the *D* matrix was used to obtain a Neighbour-Joining (NJ) population phylogenetic tree using the Phylip computer program (http://evolution.gs.washington.edu/phylip) and visualized with the online computer program Drawgram (http://www.phylogeny.fr/version2_cgi/one_task.cgi?task_type=drawgram) following a similar approach as described by Das and co-workers [21] and Gupta and co-workers [22]. In order to discern if ecological parameters (that change with population coordinates) influence the distribution patterns of the two *FY* alleles, frequencies of the two different *FY* alleles were correlated with both latitude and longitude of the population samples (determined from the centrally placed city of each zone, see Supplementary Table S1) independently by calculating the Pearson's correlation coefficient (*r*). Furthermore, since the FY*A allele has been shown to provide protection against vivax malaria in comparison to the FY*B allele [9], and epidemiology of *P. vivax* malaria varies in India, we have correlated the extent of vivax malaria for each Indian state (http://www.nvbdcp.gov.in/) with the frequencies of both the FY*A and FY*B alleles and with three *FY* genotypes by calculating the *r* values independently. Data in percent frequencies were converted through Arcsine Transformation before employing in any of the above statistical analyses.

### Ethics Statement

The Institutional ethics committee of the National Institute of Malaria Research (NIMR), New Delhi, India has approved the study and written informed consent from each blood donor has been obtained.

## Results and Discussion

In none of the 250 Indians, the C nucleotide at the −33^rd^ promoter region of the *FY* gene could be found (no FY*O allele), indicating no Duffy-negative individual in the presently analyzed samples from India. The results are not quite surprising, as some earlier studies in India employing serological techniques had identified only the tribal Indians dwelling in Andaman and Nicobar Islands and Madhya Pradesh as Duffy-negative [16], [17]. On the other hand, studies following serological techniques, no Duffy-negative individual could be found in tribes inhabiting in Rajasthan and Nagaland states [15], [18]. Non-occurrence of Duffy negative Indians, especially the three tribes presently analyzed (Juango, Bonda, Kutia Kandha inhabiting in Odisha state) indicates that the Duffy-negative Indians might be restricted to particular tribes and locations in India. More number of tribal individuals populating in different Indian states would be needed to establish the prevalence of the FY*O allele in Indians in general, and in indigenous Indians in particular. However, both the African samples employed as positive control in the present study were found to be Duffy-negative [as determined by the presence of C nucleotide instead of a T nucleotides at the −33^rd^ promoter region of the *FY* gene]; one individual with single peak of the C nucleotide (homozygous) and the other with two peaks of both T and C nucleotides (heterozygous), indicating the flawlessness of DNA sequencing in unraveling the Duffy genotypes in the present study.

For the 125^th^ nucleotide position, both the A (FY*B) and the G (FY*A) nucleotides were found to be segregated in all the six Indian zonal population samples and also in the tribes. Thus, each Indian could be characterized into one of the three genotypes at this nucleotide position; either as one of the two homozygotes (FY*A/FY*A and FY*B/FY*B) or as a heterozygote (FY*A/FY*B). In total, out of 250 Indians, 53 were homozygous for FY*B genotype; 130 were homozygous for FY*A and the rest 67 were heterozygotes (FY*A/FY*B). Similarly, both the African individuals were found to be FY*B homozygous. Distribution of the three different genotypes (FY*A/FY*A, FY*B/FY*B and FY*A/FY*B) and allele (FY*A and FY*B) frequencies in each population sample are detailed in Table 1. In general, majority of Indians (52% of the total samples) were found to be homozygous to FY*A, while Indians possessing either the FY*B homozygote or the FY*A/FY*B heterozygote were in 21.2% and 26.8%, respectively. Correspondingly, in each population sample, the FY*A homozygote was found to be highest in number than the corresponding FY*B homozygote and the heterozygote. Moreover, the observation of a comparatively lesser number of heterozygotes (FY*A/FY*B) indicates no evidence of heterozygous advantage (heterosis), except for the SI and OT population samples, where fair number of heterozygous individuals were found to be present. It is well known that heterosis is maintained by balancing selection in many natural populations for functionally relevant genes in humans [23], [24]. Absence of an adequate number of heterozygous individuals thus specifies that the *FY* gene does not evolve following the balancing selection model of natural selection in India. This observation further implicates the role of some other form of natural selection (*e.g.* positive natural selection) or population demography on the observed pattern of distribution of different alleles (and genotypes) of the *FY* gene. To this respect, observations of statistically significant deviation from the H-W equilibrium expectation in five (NI, NEI, CI, WI and EI) of the seven samples (with less number of heterozygotes, Table 1) indicate demographic disequilibrium or positive natural selection (also see below) in majority of the Indian samples. However, the observed demographic disequilibrium (excess of homozygotes) might not have been caused due to inbreeding, as each zonal population sample in the present study is composed of unrelated individuals originating from different Indian states (Supplementary Table S1). Thus, the excess of FY*A homozygote that is essentially responsible for deviation from H-W expectation in the majority of zonal population samples points towards the role of positive natural selection in maintaining the FY*A alleles in high frequency in India (also see below). Such an observation seems to be unique to India thus far, as in Brazilians (another vivax malaria endemic country), no significant departure from the H-W expectation at the *FY* gene could be observed [25]. Thus, the differences in the pattern of evolution of the *FY* gene between India and Brazil might be due to differential evolutionary pressures at the *FY* gene and variable host-parasite interaction mechanisms prevailing in the two vivax malaria eco-epidemiological settings.

**Table 1:** Details of locations and numbers of samples, average vivax malaria incidences over five years period (2007–2011), Hardy Weinberg Expectations and allele and genotype frequencies of the *FY* gene in Indians.

Locations and no. of samples	Central city	Latitude/Longitude	# *P.vivax* malaria (%)	Observed no. of genotypes			HWE (χ^2^)	Allele frequency (%)	
				FY*B/FY*B	FY*A/FY*A	FY*A/FY*B		FY*A	FY*B
North India (50)	Haridwar	29°57′N/78°10′E	97	18	22	10	17.85***	54	46
North-East India (32)	Guwahati	26°11′N/91°44′E	41	7	24	1	26.12***	77	23
East India (36)	Ranchi	23°21′N/85°20′E	49	5	22	9	4.23*	74	26
Odisha Tribes (28)	Bhubaneswar	20°16′N/85°50′E	49	5	12	11	0.73	63	37
Central India (40)	Bhopal	23°15′N/77°25′E	46	6	24	10	5.56**	72	28
West India (32)	Ahmedabad	23°02′N/77°35′E	77	9	16	7	9.35**	59	41
South India (32)	Chennai	13°05′N/80°16′E	73	3	14	15	0.01	67	33

* P<0.05,

** P<0.01,

*** P<0.001.

HWE = Hardy Weinberg Expectations.

# Average yearly proportions of *P.vivax* malaria in India. Source: www.nvbdcp.gov.in; The total number of *P. falciparum* malaria cases (as provided in the website) for each state under different zones were deducted from the total malaria cases for each year (2007–2011) to obtain the total number of *P. vivax* malaria cases The data on *P. vivax* might include a few number of *P.malariae* infections, in the EI samples.

In order to estimate genetic differentiations between different zonal populations and to perform other population genetic studies involving the *FY* gene polymorphism in India, we first calculated the frequencies of the two alleles (FY*A and FY*B) from the genotype data for each population. As expected, in all the seven samples, the frequency of the FY*A allele was higher than the FY*B, indicating high prevalence of FY*A allele in Indians (Table 1). This is interesting, as the FY*A allele (derived, conferring protection to *P. vivax* infection) [9] was found to dominate over the FY*B (ancestral, providing vivax malaria susceptibility) [26] allele in India. In order to measure genetic differentiation between population pairs at the *FY* gene, we utilized the allele frequency data and calculated the Nei's *D* [19] (Table S3) and Fisher's Exact Test. While the *D* values were utilized to reconstruct a population phylogenetic tree [21], [22] (see below); the Fisher's Exact Test statistic yielded a total of only three statistically significant cases of pair-wise genetic differentiation, always involving the NI population sample (NI *vs.* CI = 0.014; NI *vs.* EI = 0.009 and NI *vs.* NEI = 0.005), indicating that the northern Indians are well differentiated from individuals inhabiting in the central, eastern and northeastern India.

**Figure 1:**
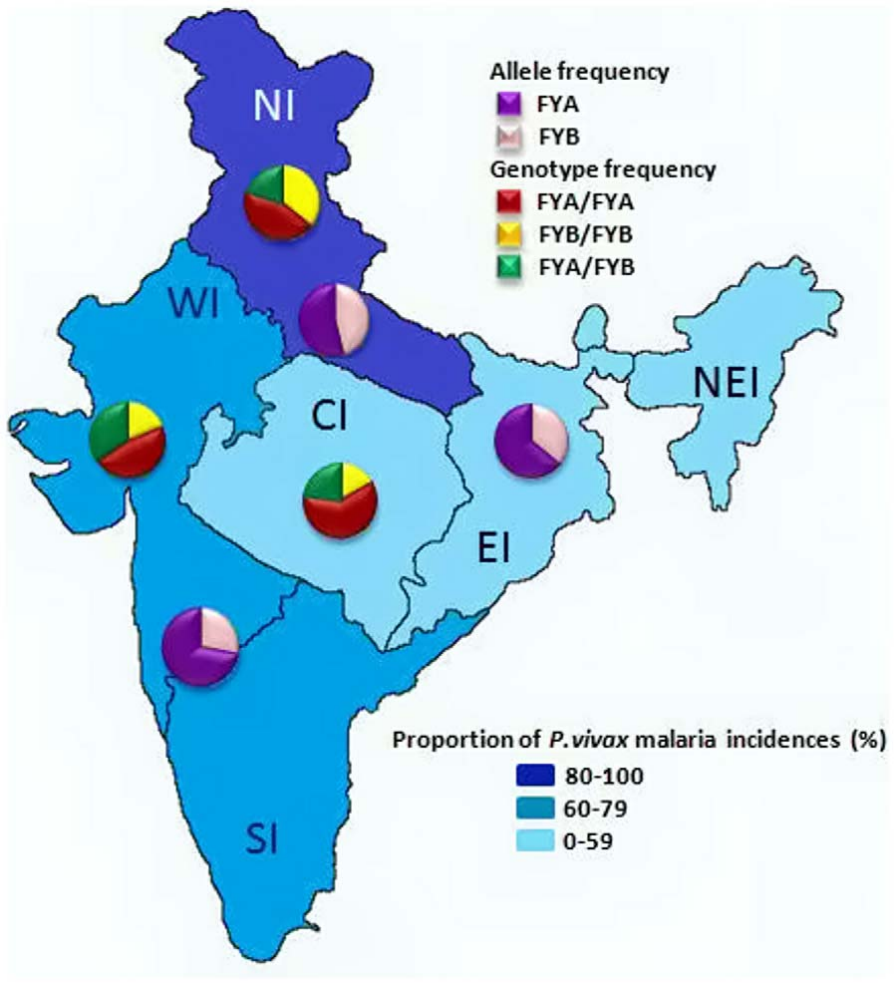
Map of India showing partitions of different zones (NI, NEI, EI. CI, WI and SI) considered for sampling of population in the present study. Three different blue colour shades represent three different vivax malaria transmission areas (high, medium and low). The frequencies of the alleles (FY*A and FY*B) and genotypes (FY*A/FY*A, FY*B/FY*B and FY*A/FY*B) were indicated as pi-charts in the three respective regions in each of these three areas.

Since the genotype frequency distribution data indicated towards the role of positive natural selection in evolution of the *FY* gene (see above), to strengthen our hypothesis we were interested to discern whether eco-climatic conditions of the population sample have influenced the frequency distribution of the two different alleles (FY*A and FY*B) of the *FY* gene in India. Although no statistically-significant correlation (with respect to the *r* values) could be obtained between the frequencies of the two alleles (FY*A and FY*B) and the latitude, statistically significant *r* values (in opposite direction for the two alleles) could be obtained with longitude (Table 2). In particular, the ancestral FY*B allele was found to be negatively correlated, and the FY*A was positively associated with the longitude in India (Table 2). Since eco-climatic conditions of different locations change with the latitude and longitude [12], the distribution patterns of the two *FY* alleles seem to follow strict eco-climatic trend in India as a result of local adaptation by natural selection. It is known that the FY*A allele have almost reached to fixation in Southeast Asia [9], and the present results indicate that its frequency decreases as we move in westward directions in India. Considering India as a part of the ancestral range of *P. vivax* [22], and the incidences of vivax malaria decreases as we move in eastward direction (Figure 1), it can be argued that the resistance to *P. vivax* infection has been well-evolved in the eastern, central and northeastern Indians as reflected by the high prevalence of the FY*A allele in those regions (Figure 1). This hypothesis is further substantiated by the fact that *P. vivax* populations from these areas in India have undergone significant population size reduction in the past [22]. However, all these regions are highly endemic to *P. falciparum* malaria [11] with high rates of mixed parasite infections [27]. Such complex malaria epidemiology in India [11], especially the decreased incidences of *P. vivax* infection over *P. falciparum* in the past years [19], presents two principal hypotheses to be tested; (i) outperformance of *P. falciparum* over *P. vivax* and (ii) evolving host resistance to vivax malaria. While testing of the first hypotheses requires separate experimental set ups, the results from the present study could be able to demonstrate evolving acquired immunity in Indians against *P. vivax* infection in general, and in the central, eastern and northeastern Indians, in particular. This proposition could possibly explain the observed declining trend of vivax malaria in these three zones in India [19], although other unseen factors might also be responsible. Furthermore, dominance of the FY*A homozygotes (75% in NEI, 60% in CI and 56% in EI) in these three zones (Central, Eastern and North Eastern) suggests that individuals homozygous to FY*A might be more capable of resisting to *P. vivax* malaria infection than the heterozygotes. Moreover, tight positive latitudinal clines for both the FY*A and FY*B homozygotes (Table 2) indicate the role of natural selection through local adaptation of these two homozygotes [28], [29]. Whether such clines are solely adaptive for both the genotypes could not be ascertained in the present study, but it might be predicted that the cline for the FY*A/FY*A genotype over latitude might be a reflection of its increasing trends towards the northern Indian populations due to local adaptation by natural selection and the cline observed for the FY*B homozygote may be historically maintained due to its ancestral background [26].

**Table 2:** Pearson's correlation coefficient (*r*) between the alleles and genotypes frequencies of the *FY* gene with vivax malaria incidences over five years period (2007–2011) and population coordinates (latitude and longitude) in India.

Alleles and genotypes of the *FY* gene		Incidences of *Plasmodium vivax* malaria in India						Population coordinates	
		2007	2008	2009	2010	2011	(2007-11)#	Latitude	Longitude
Alleles	FY*A	−0.81*	−0.83*	−0.91*	−0.95*	−0.95*	−0.92*	0.01	0.67*
	FY*B	0.83*	0.86*	0.92	0.96*	0.96*	0.94*	0.04	−0.65*
Genotypes	FY*A/FY*A	−0.09	0.04	−0.02	−0.22	−0.2	−0.2	0.68	0.21
	FY*B/FY*B	0.72*	0.89**	0.85*	0.7*	0.71*	0.79*	0.78*	−0.32
	FY*A/FY*B	0.38	0.2	0.21	0.35	0.34	0.31	−0.59	−0.41

* P<0.05,

** P<0.01.

# Five years average incidences of *P.vivax* malaria in India.

**Figure 2:**
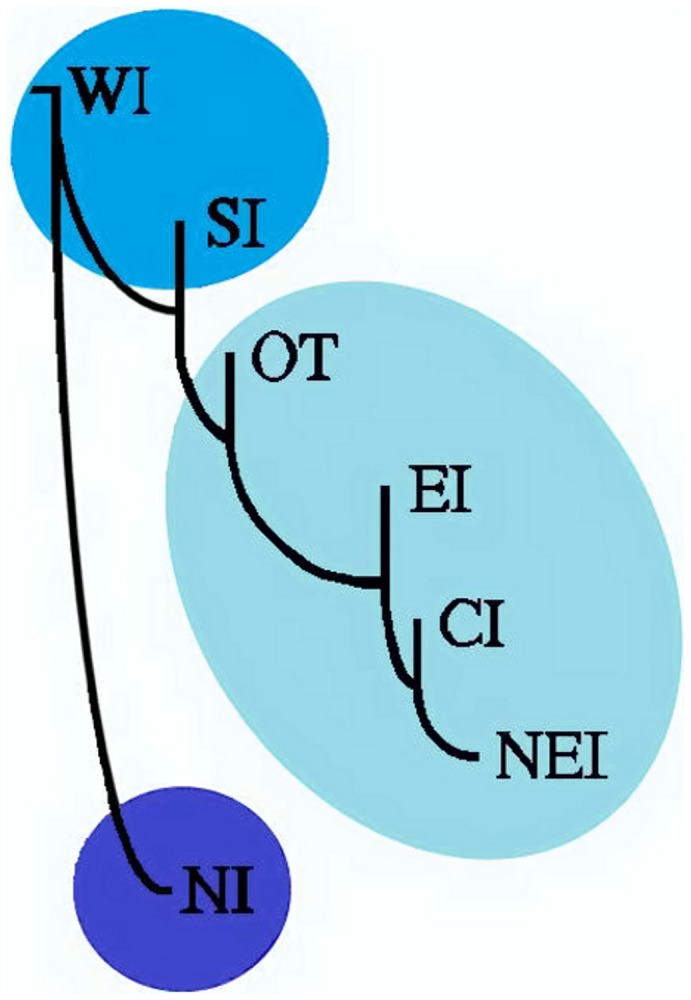
Neighbor-Joining (NJ) phylogenetic tree showing genetic interrelationship among seven different population samples based on pairwise population genetic distance matrix (Nei's *D*, Table S3). The colour codes of the three clades are same as that of the Figure 1, corresponding to differential incidences of vivax malaria (high, medium, low).

The results of the present study provide us with opportunities to test if the high prevalence of FY*A allele that is maintained by the influence of positive natural selection (see above) has, by any means, impacted the vivax malaria epidemiology in India. For this purpose, we obtained the number of vivax malaria incidences for each year separately for the last five years (2007–2011) and also five years' combined data (2007–2011) for each Indian state [courtesy; National Vector Borne Disease Control Program, (www.nvbdcp.gov.in), New Delhi, India] and collated for six zonal population samples (separately for each year and the combined data for all the five years). The five-year combined state-wise data on vivax malaria majorly fall in three different categories; high (80–100% of malaria cases due to *P. vivax* infection), medium (60–79%) and low (0–59%) (Figure 1). Accordingly, the whole of India could be divided into three distinct zones based on vivax malaria endemicity (high, medium and low) (Figure 1). Further, data on the frequency of the two alleles (FY*A and FY*B) and number of the three genotypes (FY*A/FY*A, FY*A/FY*B and FY*B/FY*B) were also assembled separately for these three zones and plotted as two different types of pie charts (for allele frequencies and for genotypes) in the respective zones (Figure 1). In addition, Pearson's correlation coefficient (*r*) values were calculated between the exact incidences of vivax malaria and the frequencies of the two *FY* alleles (FY*A and FY*B) as well as the number of three different genotypes (Table 2). As indicated in Table 2, several cases of statistically significant *r* values were obtained with such genetic and epidemiological correlation analyses, involving both the yearly as well as the five-year combined vivax malaria data in India. Interestingly, statistically significant correlations for both the FY*A and FY*B alleles were observed over all the five years (2007–2011), and also for the combined five year data. However, the trends were in opposite directions (negative correlation for FY*A and positive for FY*B) (Table 2). For the genotypes, the FY*B homozygote presented statistically significant positive correlations with vivax malaria cases in all the five years independently (and also with the five-year combined data), corroborating the fact that individuals of this particular genotype (FY*B/FY*B) may be highly susceptible to vivax malaria. However, no such association over the years could be observed for either the FY*A/FY*A homozygote or the FY*A/FY*B heterozygote (Table 2). However, considering the FY*A genotype lowers the risk to vivax malaria than the FY*B allele [9], and field epidemiological data on vivax malaria in India reveal negative association with the frequency distribution of the FY*A alleles and positive association with the FY*B alleles (see above), it can be ascertained that the FY*A allele might be playing some role in conferring protection to vivax malaria, and is maintained under positive natural selection in India. Whether such an association exists in other vivax malaria endemic countries remains to be seen, as this type of study has never been conducted in any other malaria epidemiological setting yet. On the contrary, the observed statistically significant positive association between vivax malaria and FY*B allele frequency was mainly due to high prevalence of the FY*B homozygote in places where vivax malaria incidences are high (*e.g.* NI population sample) (Table 1). To this respect, it was interesting to know if genetic relatedness among different Indian zonal samples fits well with the observed differential frequency distribution of the FY*A and FY*B alleles by reconstruction of a NJ population phylogenetic tree (Figure 2) from genetic distance matrix [21], [22]. Interestingly, the NJ tree produced three different clades (Figure 2), exactly representing the three distinct zones based on differential incidences of vivax malaria (high, medium and low) (Figure 1). The northern Indian (NI) sample was clearly separated from the rest six population samples in the NJ tree (Figure 2), suggesting the fact that this population sample (with the highest incidences of both vivax malaria and FY*B alleles) is in fact genetically distinct from the rest of six Indian population samples. This observation further corroborated with (i) the observations on the statistically significant Fisher's Exact Test between the NI and three other population samples (see above) and (ii) the partitioning of whole of India based of differential incidences of vivax malaria (Figure 1). The results from a diverse array of population genetic analyses thus point towards correspondence between the genetic (*FY* gene) and epidemiological (incidences of *P. vivax* infection) aspects in Indians.

In conclusion, the results of the present population genetic study indicate the role of natural selection in the variable distribution of two different alleles of the *FY* gene (FY*A and FY*B) in Indians. The significant departure from H-W expectation in five out of seven samples justifies this contention. In addition, a clear-cut negative correlation with vivax malaria epidemiology in Indians with the FY*A allele frequency distribution points towards the FY*A homozygote genotype being under strong influence of positive natural selection. In view of the fact that the derived FY*A allele confers resistance to vivax malaria [9], its tight negative correlation with incidences of vivax malaria indicates evolving resistance to vivax malaria in Indians. However, the prevalence of FY*B allele in all zones (although in a lower frequency than the FY*A allele) suggests that Indians are still susceptible to *P. vivax* infection. Since it is argued that the ideal populations to test PVDBPII-based vaccine are with genetic background where combinations of FY*A and FY*B alleles exist [9], India can be the model field for trial of such a vaccine against *P. vivax* infection. Considering both the falciparum and vivax malaria are prevalent in almost all over India [11], [19] and the *FY* polymorphisms have been subjected to malarial selection shortly before known *P. falciparum* protective mutation [30], [31], comparative genetic studies of genes responsible for susceptibility to both falciparum and vivax malaria in common populations could not only inform evolution of host-parasite interaction mechanisms in malaria but also help in devising novel control measures for both forms of malaria in India.

## Supporting Information

Figure S1 **Schematic diagram of the of 1096 bp DNA fragment of the Duffy gene.**
(TIF)

Table S1 **Details of the sample location, size and vivax malaria proportions for each Indian state that are used to form six different zones for the present study.**
(RTF)

Table S2 **Description of primer sequences and annealing temperatures for PCR amplification and sequencing of the 1096 bp DNA fragment.**
(RTF)

Table S3 **Pairwise-population genetic distance as calculated using Nei's genetic distance (Nei's** ***D*** **, 1972).**
(RTF)
