# Supplementary figures and images for: Natural Selection Mediated Association of the Duffy (FY) Gene Polymorphisms with Plasmodium vivax Malaria in India
Source: PLoS One. 2012 Sep 21;7(9):e45219. doi: 10.1371/journal.pone.0045219 (PMC3448599; doi:10.1371/journal.pone.0045219)

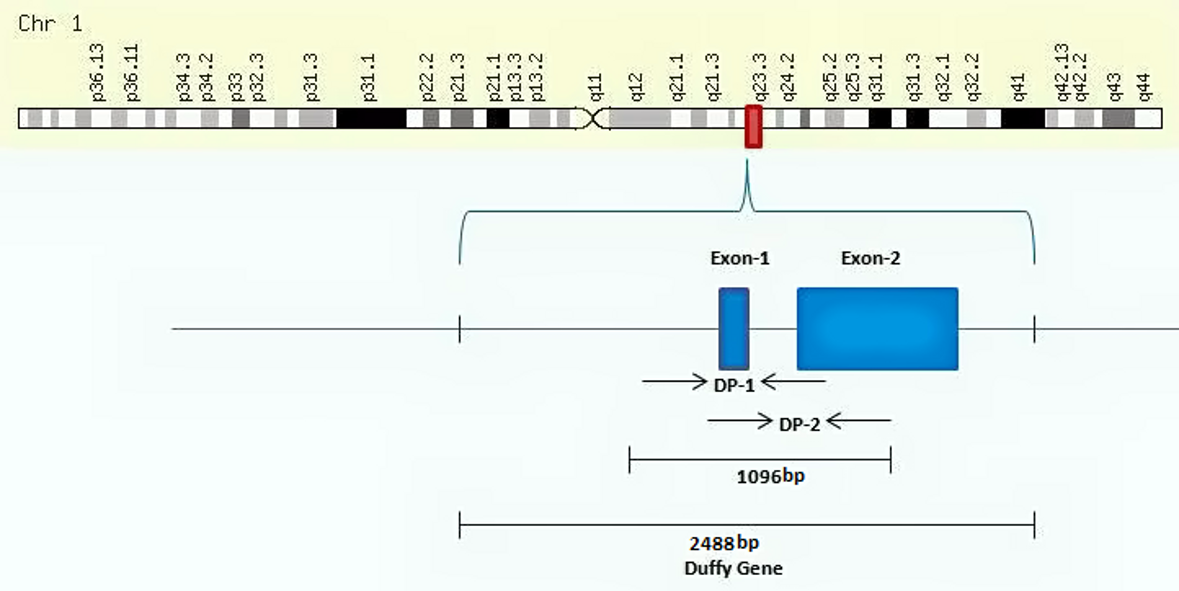

Supplement: Figure S1 — Schematic diagram of the of 1096 bp DNA fragment of the Duffy gene. (TIF) [file pone.0045219.s001.tif]
