# Supplementary material for: Natural Selection Mediated Association of the Duffy (FY) Gene Polymorphisms with Plasmodium vivax Malaria in India
Source: PLoS One. 2012 Sep 21;7(9):e45219. doi: 10.1371/journal.pone.0045219 (PMC3448599; doi:10.1371/journal.pone.0045219)
Supplement: Table S1 — Details of the sample location, size and vivax malaria proportions for each Indian state that are used to form six different zones for the present study. (RTF) [file pone.0045219.s002.rtf]

Table S1
Different zones of India	States	Sample Size	Population Coordinates	Average Plasmodium vivax malaria proportions (%)	
		Total	Female	Male	Latitudinal	Longitudinal	2007	2008	2009	2010	2011	2007-11	
North India (NI)	Jammu & Kashmir	6	2	4	34005'N	74047'E	98	97	98	97	97	97	
	Himachal Pradesh	7	2	5	31006'N	77010'E							
	Uttaranchal	11	2	9	29057'N	78010'E							
	Punjab	6	2	4	31064'N	74086'E							
	Haryana	10	6	4	28025'N	77018'E							
	Uttar Pradesh	10	3	7	26085'N	80092'E							
North East India (NEI)	Sikkim	3	1	2	27033'N	88062'E	50	43	0.37	0.38	0.38	41	
	Assam	3	0	3	26011'N	91044'E							
	Meghalaya	2	1	1	25056'N	91088'E							
	Nagaland	5	0	5	25067'N	94012'E							
	Manipur	8	3	5	24082'N	93095'E							
	Mizoram	3	1	2	23042'N	92094'E							
	Tripura	2	0	2	23050'N	91005'E							
	Arunachal Pradesh	6	2	4	27006'N	93037'E							
West India (WI)	Rajasthan	8	1	7	26050'N	80050'E	65	66	0.82	0.86	0.87	77	
	Gujarat	6	0	6	23002'N	72035'E							
	Maharashtra	8	2	6	18096'N	72082'E							
	Goa	10	1	9	15025'N	73005'E							
Central India (CI)*	Madhya Pradesh	34	6	28	23015'N	77025'E	45	42	49	45	48	46	
	Chhattisgarh	3	3	0	21014'N	81038'E							
	Southern Uttar Pradesh	1	1	0	25028'N	82095'E							
	North Eastern Maharashtra	2	0	2	21007'N	79027'E							
East India (EI)	Orissa	9	1	8	20027'N	85084'E	54	46	43	50	50	49	
	Jharkhand	7	3	4	23021'N	85020'E							
	Bihar	12	4	8	25061'N	85014'E							
	West Bengal	8	1	7	22030'N	88030'E							
Odisha Tribes
(OT)	Juango	6			20016'N	85050'E							
	Bonda	11											
	Kutia Kandha	11											
South India (SI)	Karnataka	4	1	3	12097'N	77056'E	82	61	63	80	79	73	
	Kerala	10	2	8	08017'N	76041'E							
	Tamil Nadu	12	2	10	13005'N	80016'E							
	Andhra Pradesh	6	0	6	17036'N	78047'E							
Total		250	53	169									

*Include parts of Uttar Pradesh (southern) and Maharashtra (northeastern) states. However, vivax malaria proportions of these two states are not included in this zone. For Uttar Pradesh and Maharashtra, the vivax malaria data were included in the NI and WI, respectively.
