# Supplementary material for: Natural Selection Mediated Association of the Duffy (FY) Gene Polymorphisms with Plasmodium vivax Malaria in India
Source: PLoS One. 2012 Sep 21;7(9):e45219. doi: 10.1371/journal.pone.0045219 (PMC3448599; doi:10.1371/journal.pone.0045219)
Supplement: Table S2 — Description of primer sequences and annealing temperatures for PCR amplification and sequencing of the 1096 bp DNA fragment. (RTF) [file pone.0045219.s003.rtf]

Table S2. 

Size of fragment	Primer Sequences	Annealing Temperatures	
630bp	F-5' TTTCCTGAGTGTAGTCCCAACC 3'        
R-5' AAGGTCTCTGCAGGAGTCAGAT 3'	640C	
599bp	F-5' CCTTCTGACCTTGCACTGCT 3'    
R-5' CAGAGCTGCGAGTGCTACCT 3'  	620C	
