# Supplementary material for: Natural Selection Mediated Association of the Duffy (FY) Gene Polymorphisms with Plasmodium vivax Malaria in India
Source: PLoS One. 2012 Sep 21;7(9):e45219. doi: 10.1371/journal.pone.0045219 (PMC3448599; doi:10.1371/journal.pone.0045219)
Supplement: Table S3 — Pairwise-population genetic distance as calculated using Nei's genetic distance (Nei's D , 1972). (RTF) [file pone.0045219.s004.rtf]

Table S3.  
==========================================================
Pop        NI           CI            WI            EI            NEI           SI            OT         
==========================================================
NI          ****    
CI         0.0676      ****    
WI       0.0056    0.0337      ****    
EI         0.0668    0.0000    0.0331      ****    
NEI      0.1078    0.0043    0.0627    0.0045      ****    
SI         0.0190    0.0144    0.0039    0.0140    0.0346      ****    
OT       0.0198    0.0137    0.0043    0.0133    0.0335    0.0000      ****    
==========================================================
